# A review of -multidrug-resistant Enterobacteriaceae in a neonatal unit in Johannesburg, South Africa

**DOI:** 10.1186/s12887-019-1709-y

**Published:** 2019-09-07

**Authors:** Daynia E. Ballot, Rosella Bandini, Trusha Nana, Noma Bosman, Teena Thomas, Victor A. Davies, Peter A. Cooper, Mervyn Mer, Jeffrey Lipman

**Affiliations:** 1 Neonatal Unit, Department of Paediatrics and Child Health, University of the Witwatersrand and Charlotte Maxeke Johannesburg Academic Hospital, Johannesburg, South Africa; 2 Infection control, Charlotte Maxeke Johannesburg Academic Hospital, Johannesburg, 2196 South Africa; 3 Critical Care Infection Collaboration, Witwatersrand, South Africa; 4 Department of Clinical Microbiology and Infectious Diseases, School of Pathology of the National Health Laboratory Services and University of Witwatersrand, Witwatersrand, South Africa; 5 Department of Critical Care, University of the Witwatersrand, Witwatersrand, South Africa; 6 The University of Queensland, Brisbane, Australia

**Keywords:** Neonatal Sepsis, Enterobacteriaceae, Carbapenem-resistant, *Klebsiella pneumoniae*

## Abstract

**Background:**

Multi-drug resistant organisms are an increasingly important cause of neonatal sepsis.

**Aim:**

This study aimed to review neonatal sepsis caused by multi-drug resistant Enterobacteriaceae (MDRE) in neonates in Johannesburg, South Africa.

**Methods:**

This was a cross sectional retrospective review of MDRE in neonates admitted to a tertiary neonatal unit between 1 January 2013 and 31 December 2015.

**Results:**

There were 465 infections in 291 neonates. 68.6% were very low birth weight (< 1500 g). The median age of infection was 14.0 days. Risk factors for MDRE included prematurity (*p* = 0.01), lower birth weight (*p* = 0.04), maternal HIV infection (*p* = 0.02) and oxygen on day 28 (*p* < 0.001). The most common isolate was *Klebsiella pneumoniae* (66.2%). Total MDRE isolates increased from 0.39 per 1000 neonatal admissions in 2013 to 1.4 per 1000 neonatal admissions in 2015 (*p* < 0.001). There was an increase in carbapenem-resistant Enterobacteriaceae (CRE) from 2.6% in 2013 to 8.9% in 2015 (*p* = 0.06). Most of the CRE were New Delhi metallo—β lactamase- (NDM) producers.

The all-cause mortality rate was 33.3%. Birth weight (*p* = 0.003), necrotising enterocolitis (*p* < 0.001) and mechanical ventilation (*p* = 0.007) were significantly associated with mortality. *Serratia marcescens* was isolated in 55.2% of neonates that died.

**Conclusions:**

There was a significant increase in MDRE in neonatal sepsis during the study period, with the emergence of CRE. This confirms the urgent need to intensify antimicrobial stewardship efforts and address infection control and prevention in neonatal units in LMICs. Overuse of broad- spectrum antibiotics should be prevented.

## Background

Sepsis remains a major cause of morbidity and mortality in preterm infants [1]. There has been a significant increase in neonatal sepsis caused by multi-drug resistant organisms (MDRO) in the past decade [1, 2]. More than half the organisms causing bloodstream infections (BSI) in a neonatal unit in Johannesburg, South Africa were due to MDRO [3]. In a recent report from Jordan, two thirds of organisms causing neonatal sepsis were MDRO and most of the gram-negative organisms were extended-spectrum beta-lactamase (ESBL) producers [1]. Many preterm infants are colonised with MDRO - more than half the *Klebsiella pneumoniae* and *Escherichia coli* isolated from a group of preterm infants in Malaysia were MDRO [4].

Infections with multi-drug resistant gram-negative organisms, especially Enterobacteriaceae, are of concern in preterm infants. Neonatal sepsis caused by these pathogens is increasing and there are limited choices available for treatment [5]. Infections with multi-drug resistant Enterobacteriaceae (MRDE) are associated with poor outcome and high case fatality rates, especially in low and middle income countries (LMIC) [5]. Mechanisms of antibiotic resistance in Enterobacteriaceae include production of ESBLs or carbapenemases [5]. There are recent reports of colistin-resistant Enterobacteriaceae in neonates [4].

Carbapenem-resistant organisms were already described as a cause of neonatal septicaemia in India in 2007 [6]. It is not clear whether patterns of ESBL and carbapenem-resistant Enterobacteriaceae (CRE) reflect national resistance patterns or are specific to the neonatal units. The predominant CRE strain in Asia and the West Pacific is New Delhi metallo-beta-lactamase (NDM − 1), whereas that in Europe and the USA is *Klebsiella pneumoniae* carbapenemase (KPC) [5]. There is limited information on CRE in Africa. There is a report from Morocco where the predominant strain was OXA β-lactamase, i.e. OXA-48 carbapenemase [7].

{Magiorakos, 2012 #2052} NDM and KPC (KPC-2) were first described in adult patients in Johannesburg in 2011 [8]. The aim of this study is to describe the patterns of MDRE, including CRE, in a neonatal unit in Johannesburg, South Africa.

## Subjects and methods

This is a retrospective descriptive cross-sectional study. All newborn neonates admitted to the neonatal unit between 01 January 2013 and 31 December 2015 were eligible for inclusion. The study group included all neonates with culture proven blood stream infection (BSI) caused by MDRE. A control group of 30% of all neonates without infection admitted to the neonatal unit during the study period was randomly generated from the neonatal database using SPSS IBM 24. Subjects were identified through the laboratory information system of the National Health Laboratory Service (NHLS). Patient characteristics were obtained from the neonatal computer database. Information was obtained from hospital records on discharge of each neonatal patient and was entered into a computerised database for the purpose of quality control. Data was managed using Research Electronic Data Capture (REDCAP), hosted by the University of the Witwatersrand [9]. Maternal information, demographic and clinical characteristics, as well as survival to hospital discharge, were described for each patient. Causative organisms and their antimicrobial sensitivity patterns were described. Organism identification and antimicrobial susceptibility testing was done on the Vitek 2® (bioMerieux, Marcy-I’Etoile, France). Vitek 2 breakpoint interpretation was based on the Clinical and Laboratory Standards Institute (CLSI) guidelines. Isolates were characterised as CRE based on carbapenem Etest® (bioMerieux, Marcy-I’Etoile, France) minimum inhibitory concentration (MIC) testing. Colistin broth micro-dilution testing was not performed and hence colistin susceptibility rates cannot be reported for all isolates. Multiplex PCR for the carbapenemase genes (for *bla*NDM, *bla*KPC, *bla*OXA-48 and its variants, *bla*GES, *bla*IMP and *bla*VIM; LightMix Modular kits, Roche Diagnostics, Basel, Switzerland) was performed on a subset of the CRE isolates. Typing of isolates was not performed.

### Statistical analysis

IBM SPSS 24 was used to analyse the data.. Maternal and neonatal characteristics were described for each patient (not bacterial isolate). Microbiological information (resistance patterns, isolates over time) was analysed for each bacterial isolate. Mean and standard deviation or median and range, were used to describe central tendency in continuous variables, depending on the distribution of the data. Categorical variables were described using frequency and percentages. Only valid cases were analysed for each variable (i.e. missing cases were excluded). Two comparisons were performed. Firstly, survivors and non- survivors within the MDRE group were compared to determine risk factors for mortality. Secondly, the MDRE group and control group were compared to establish associations with MDRE infection. Frequencies were compared using Chi Square analysis, while unpaired t tests were used to compare continuous variables, as the data was normally distributed. A *p* value of 0.05 was considered to be statistically significant. Adjusted odds ratios were determined through binary logistic regression for significant associations with mortality and MDRE infection respectively.

### Ethics

Ethics clearance was obtained from the Human Research Ethics Committee of the University of the Witwatersrand (Certificate M 151108). Permission was obtained to access the Laboratory information system from the NHLS.

### Definitions

Early-onset sepsis (EOS) was defined as culture proven sepsis within the first 72 h of life, while late onset sepsis (LOS) was referred to as culture proven sepsis after 72 h of life [1]. Multidrug resistance was defined as the isolate being non-susceptible to ≥1 agent in ≥3 antimicrobial categories [10]. The presence of resistance to third generation cephalosporins was used as a marker for ESBL production. The presence of cefoxitin resistance was used as a marker for Amp C beta-lactamase production. Necrotising enterocolitis (NEC) was defined as modified Bell’s stages 2 or 3 [11]. Resuscitation at birth was defined as the need for bag mask ventilation. “Outborn” referred to all neonates born outside the study hospital. Very low birth weight indicated neonates with a birth weight below 1500 g. Mortality was defined as all-cause mortality during hospitalization.

## Results

### Characteristics associated with MDRE sepsis

There were a total of 465 MDRE infections in 291 neonates and 2146 control neonates without infection. The comparison between neonates with MDRE and controls is shown in Table 1. Control neonates weighed significantly more at birth than those neonates with MDRE sepsis 1878 g (SD 956) vs 1438 g (SD 660) (*p* < 0.001). Neonates with MDRE sepsis were significantly more preterm than controls − 30.4 weeks (SD 4.0) vs 32.9 weeks (SD 4.8) (p < 0.001) The all-cause mortality rate was higher in MDRE neonates than controls − 97/291 (33.3%) vs- 49/2146 (2.2%)) (*p* < 0.001). Most of the neonates with MDRE sepsis (199/291; 68.6%) were very low birth weight. The median age at presentation with MDRE was 14.0 days (IQR 20). Although more than one third of neonates with MDRE sepsis were HIV exposed, there was only one neonate (1/108; 0.9%) with a positive HIV polymerase chain reaction at birth. Adjusted odds ratios for those variables significantly associated with MDRE are shown in Table 2. Prematurity, lower birth weight, maternal HIV infection and oxygen on day 28 were all associated with MDRE infection.

**Table 1:** Characteristics associated with multi-drug resistant Enterobacteriaceae infection in neonates

Variable	Babies with MDRE sepsis n/N (%)	Babies without sepsis n/N (%)	*p* value
Inborn	196/288 (68.1)	1716/2096 (81.9)	< 0.001
Attended antenatal care	188/254 (74.0)	1663/1971 (84.4)	< 0.001
Maternal chorioamnionitis	7/238 (2.9)	47/1875 (2.5)	0.689
Maternal HIV	108/279 (38.7)	600/2009 (29.9)	0.003
Vaginal Delivery	134/261 (51.3)	902/2076 (43.4)	0.016
Male	157/291 (54.0)	1113/2135 (52.1)	0.697
Multiple gestation	44/284 (15.5)	267/2078 (12.8)	0.216
Resuscitation at birth	112/269 (41.6)	451/2086 (21.6)	< 0.001
Oxygen on day 28	118/262 (45.0)	161/1957 (8.2)	< 0.001
Nasal CPAP	168/191 (87.9)	961/2146 (44.8)	< 0.001
Mechanical ventilation	85/196 (43.4)	791/2101 (37.6)	0.115
Necrotising enterocolitis (Grade 2 and 3)	61/288 (21.2)	164/2080 (7.9)	< 0.001
Surgery (excluding necrotising enterocolitis)`	45/284 (15.8)	71/2065 (3.4)	< 0.001

**Table 2:** Adjusted odds ratios for characteristics associated with multi-drug resistant Enterobacteriaceae infection in neonates

Characteristic	Odds ratio	95% confidence interval	*P* value
Maternal HIV	1.60	1.076–2.303	0.02
Oxygen on day 28	5.855	3.976–8.621	< 0.001
Birth weight	0.998	0.997–0.999	0.01
Gestational age	0.881	0.794–0.977	0.04

### Bacterial isolates

There were 41/465 (8.8%) of EOS caused by MDRE. Most neonates (195/291; 67%) had a single episode of infection, 18.2% (53/291) had two episodes of infection and 14.8% (43/291) had three or more episodes. The most common isolate was *Klebsiella pneumoniae* (308/465; 66.2%), followed by *Enterobacter cloacae*. (49/465; 10.5%), *Escherichia coli* (45/465; 9.6%); *Serratia marcescens* (29/465; 6.2%), *Klebsiella* spp. (*Klebsiella* spp. other than *K. pneumoniae*) (27/465; 5.8%), *Proteus mirabilis* (3/465, 0.65%), *Citrobacter freundii* (2/465, 0.43%), *Citrobacter koseri* (1/465, 1.2%) and *Salmonella* spp. (1/465, 1.2%).

Resistance patterns of all isolates are shown in Table 3. High rates of resistance to ampicillin and amoxicillin -clavulanic acid were observed across most of the Enterobacteriaceae. Seventy-one percent (330/463) and 23% (107/456) of all MDRE isolates were ESBL- and Amp C β-lactamase-producers, respectively. Of the 29% of MDRE isolates that were not ESBL producers, 40% (53/133) were Amp C-producers. Non-ESBL, non-Amp C-producing isolates (80/133) remained susceptible to cefepime. Eighty-two percent (369/452) of MDRE isolates retained susceptibility to ciprofloxacin. Both imipenem (426/448) and meropenem (439/461), showed a high susceptibility rate of 95%. Amikacin displayed a slightly higher susceptibility rate at 96% (433/448).

**Table 3:** Antimicrobial resistance patterns in different isolates (%)

Antimicrobial agent	*K.Pneumonia*	Klebsiella sp.	*Enterobacter*	*E.Coli*	*Serattia*	Other
Ampicillin/Amoxil	98,7	100	85,7	84,4	84,2	100
Amox-Clavulanate	75,9	55,5	95,9	26,6	100	14,2
Piperacillin-tazobactam	39,4	25,9	22,4	6,6	14,2	14,2
Cefotaxime	89,8	62,9	31,9	20	27,5	14,2
Ceftazidime	90,5	59,5	28,5	20	27,5	14,2
Cefepime	89,2	53,8	12,2	17,7	24,1	0
Ciprofloxacillin	22,9	18,5	0	0	0	0
Ertapenem	4,3	0	2,1	0	0	28
Imipenem	6,1	3,7	0	0	0	28,5
Meropenem	6,5	3,8	0	0	0	14,2
Amikacin	4,9	5	0	2,2	0	25
Tobramycin	66,6	50	4	25,9	25	30

**Fig. 1:**
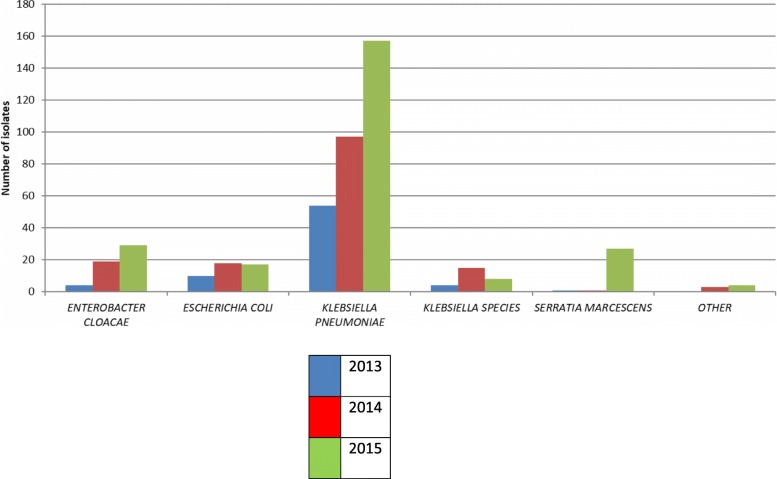
Multi drug resistant bacterial isolates by year

**Fig. 2:**
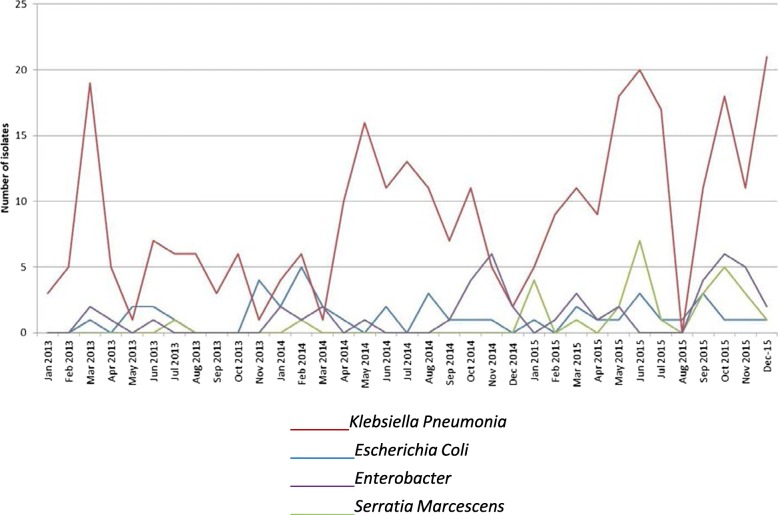
Multi-drug resistant Enterobacteriaceae bacterial isolates by month

The variation in annual MDRE isolates is shown in Fig. 1. Total MDRE isolates increased from 0.39 per 1000 neonatal admissions in 2013 to 1.4 per 1000 neonatal admissions in 2015 (*p* < 0.001). Monthly variation in isolates of *K. pneumoniae*, *E. cloacae, S. marcescens* and *E. coli* is shown in Fig. 2.

### Carbapenem resistant isolates

**Fig. 3:**
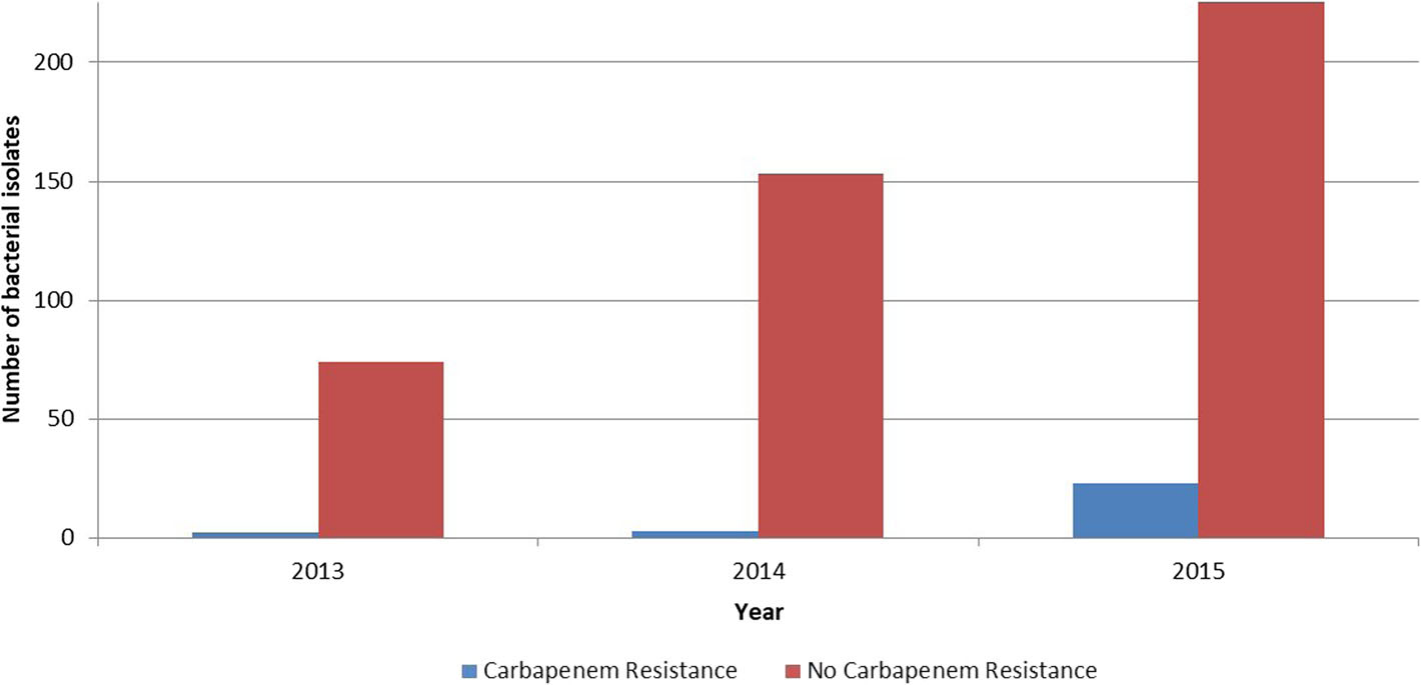
Carbapenem resistance by year

There were 26 CREs isolated from 24 neonates shown in Table 4. There was one isolate each of *C. freundii* and *Klebsiella oxytoca* and two *E. cloacae*. The remaining 22 isolates were *K. pneumoniae*. CRE isolates per year are shown in Fig. 3. There was an increase in CRE from 2.6% in 2013 to 8.9% in 2015 (*p* = 0.06). One *Klebsiella pneumoniae* isolate produced Verona Integron-Mediated Metallo-β-lactamase (VIM) and fourteen produced NDM. The *K. oxytoca* produced VIM and the *C. freundii* produced NDM. One isolate was negative for CPE genes and 8 isolates were not tested. Ninety-two percent (24/26) and 62% (16/26) of the CRE isolates were ciprofloxacin and amikacin susceptible, respectively.

**Table 4:** Carbapenem resistant Enterobacteriaceae determinants and susceptibility profiles

Number	Organism	Carbapenemase Gene	Antibiotic Susceptibility				
			Amikacin	Ciprofloxacin	Ertapenem	Meropenem	Imipenem
1.	*K. pneumoniae*	*NT	**R	***S	R	R	S
2.	*K. pneumoniae*	NT	****I	S	R	I	S
3.	*K. pneumoniae*	NDM	R	S	NT	R	R
4.	*K. pneumoniae*	NDM	R	R	R	R	R
5.	*K. pneumoniae*	NDM	S	S	NT	R	R
6.	*K. pneumoniae*	NDM	R	S	R	R	R
7.	*K. pneumoniae*	VIM	S	S	S	R	R
8.	*K. pneumoniae*	NT	S	S	R	R	R
9.	*K. pneumoniae*	NT	S	S	R	R	R
10.	*K. pneumoniae*	NDM	S	S	NT	R	R
11.	*K. pneumoniae*	NDM	S	S	R	R	R
12.	*K. pneumoniae*	NDM	R	S	NT	R	R
13.	*K. pneumoniae*	NDM	R	S	NT	R	R
14.	*K. pneumoniae*	NDM	S	S	NT	R	R
15.	*K. pneumoniae*	NDM	S	S	R	R	R
16.	*K. pneumoniae*	NDM	R	S	NT	R	R
17.	*K. pneumoniae*	NDM	R	S	R	R	R
18.	*K. pneumoniae*	NDM	S	S	NT	R	R
19.	*K. pneumoniae*	NDM	S	S	NT	R	R
20.	*K. pneumoniae*	NT	S	S	R	R	R
21.	*K. pneumoniae*	NEG	S	S	R	R	R
22.	*K. pneumoniae*	NT	S	R	R	S	I
23.	*K. oxytoca*	VIM	S	S	NT	R	R
24.	*E. cloacae*	NT	S	S	S	S	R
25.	*E. cloacae*	NT	S	S	R	S	S
26.	*C. freundii*	NDM	R	S	R	R	R

NT- not tested

**R- resistant

***S- susceptible

****I- intermediately-susceptible

The susceptibility to Amikacin and/or Ciprofloxacin allows these antibiotics to be considered as treatment options either as (1) monotherapy or (2) in combination with each other or (3) in combination with a carbapenem, which spares colistin use

### Mortality in the MDRE neonates

All-cause mortality in the neonates with MDRE sepsis was 33.3% (97/291). Adjusted odds ratios for variables significantly associated with mortality were birth weight (OR 0.997; 95%CI 0.996–0.999, *p* = 0.003), NEC (OR 4.644; 95%CI 2.012–1-.715, *p* < 0.001) and mechanical ventilation (2.496; 95%CI 1.282–4.860, *p* = 0.007). Mortality rate by isolate is shown in Table 5. The highest mortality rate was seen in neonates with *S. marcescens* BSI (55.2%) (*p* < 0.017). Neither ESBL production nor carbapenem resistance was significantly associated with mortality.

**Table 5:** Mortality by bacterial isolate

Bacteria	Total	Died	% Mortality
*Enterobacter cloacae*	46	20	43.5
*Escherichia coli*	42	17	40.5
*Klebsiella pneumoniae*	306	89	29.1
*Klebsiella spp*	25	5	20.0
*Serratia marcescens*	29	16	55.2
Other	6	2	33.0

## Discussion

This is the first report on the increase in MDRE, in particular CRE, in neonates in South Africa. The most common isolate was *K. pneumoniae.* These results are in keeping with other reports from LMICs where *Klebsiella* spp. have also been the predominant pathogen causing neonatal sepsis [12, 13]. The Study for Monitoring Antimicrobial resistance (SMART), which studied bacterial resistance patterns globally between 2002 to 2011 from isolates in urinary tract infections and intra-abdominal sepsis, reported an increase in ESBLs in all continents except Africa [14]. Unfortunately, this is no longer the case - ESBL isolates are not uncommon in Africa. A report form Burkina Faso found that almost 60% of Enterobacteriaceae were ESBL producers, [15]. while more than half of all *Klebsiella i*solates in South Africa were reported to produce ESBLs [16]

In the present study, there was a marked increase in all MDRE over the study period, but particularly in isolates of *Klebsiella* spp., which more than doubled. This increase in MDRE in neonates is in keeping with global trends [2, 5]. Carbapenem-resistant *K. pneumoniae* was first reported in 2008 in neonates in Karachi, Pakistan [2]. and by 2011, 72% of *K. pneumoniae* isolates were resistant to carbapenems. The first case of carbapenem-resistant *K. pneumoniae* in a neonate in South Africa was described in 2015 in KwaZulu Natal [17], while invasive CRE was isolated in paediatric patients in Cape Town in 2012 [18]. Neonatal CRE was first noted in the present study in 2013 and by 2015, 7.3% of the MDRE isolates were carbapenem resistant.

Factors associated with MDRE sepsis in the present study included prematurity, oxygen on day 28 of life and lower birth weight. This is in agreement with other reports where prematurity, low birth weight, prolonged hospitalization, surgical procedures, mechanical ventilation and use of invasive devices were reported as risk factors for MDRE [19–22]. However, the risk factors for all late onset neonatal sepsis include lower birth weight and gestational age, necrotising enterocolitis and bronchopulmonary dysplasia [23]. In order to determine risk factors that are specific for MDRE infection, a control group of neonates with late onset sepsis without MDRE organisms should be used, rather than neonates without sepsis. The case fatality rate in the current study was 33.3% which is slightly higher than that reported from a review of MDRE in Taiwan [24]. Mortality in the present study was significantly associated with lower birth weight, NEC and mechanical ventilation.

Most of the MDRE isolates in the current study were resistant to the penicillins and cephalosporins; the most common mechanism of resistance being production of ESBLs, particularly in *Klebsiella* spp. The frequency of ESBL producers in the current study resulted in widespread use of carbapenems (usually meropenem) for empiric treatment of presumed sepsis in neonates. This compounds the problem of resistant organisms as widespread use of broad-spectrum antibiotics is an important mechanism in the development and spread of MDRE [5, 20, 21, 25]. Previous exposure to carbapenems is a risk factor for subsequent CRE [18]. Treatment options for confirmed MDRE sepsis were also limited. Fluoroquinolones and aminoglycosides were not frequently used, due to significant side effects. Cefepime remains a treatment option for the isolates that were non-ESBL and non-Amp C producers. Meropenem was the appropriate antibiotic choice in the majority of isolates. Several of the CRE isolates were susceptible to Amikacin and/or Ciprofloxacin. As a result, these antibiotics could be considered as treatment options either as (1) monotherapy or (2) in combination with each other or (3) in combination with a carbapenem. This would result in sparing Colistin use. The emergence of colistin resistance in neonatal sepsis has been reported in other studies [5, 24]. The lack of appropriate colistin susceptibility testing precludes the determination of the presence colistin resistance in this study. Previous reports have suggested a seasonal incidence in *Klebsiella* infections in neonates [2]. Although there was marked variation in the number of MDRE isolate (including *Klebsiella* spp.*)* in the current study, there was no evidence of a seasonal variation.

The mortality rate was highest in neonates infected with *S. marcescens* in the present study. *S. marcescens* is an important nosocomial pathogen, especially in neonatal intensive care units (NICU) where several outbreaks have been described globally. It causes serious infections, including bacteremia, pneumonia**,** urinary tract infections and meningitis with significant morbidity and mortality rates among newborns. Risk factors for acquisition of nosocomial infections caused by *S. marcescens* in NICUs are low birth weight, long duration of hospitalization and receiving of critical care [26]. Although we found a higher mortality rate in *S. marcescens* infections, we did not analyze possibly confounding variables such as the severity of illness related to prematurity in these infants.

Prevention of MDRE infections includes screening for colonization, antibiotic stewardship, and stringent infection control practices including, hand hygiene practices, use of appropriate personal protective equipment, and decreased use of invasive devices [20, 25]. The importance of the neonatal gut microbiome has been underestimated. A recently published systematic review showed that supplementation with probiotics reduced the incidence of LOS in preterm infants [27]. Many neonates colonized with MDRE develop infection with the same organism and the presence of components of the gut normal flora, such as *Lactobacillus plantarum* and *Bifidobacterium breve,* can have a protective effect by exhibiting antimicrobial activity against gut pathogens [25]

### Limitations of the study

This was a retrospective review of an existing database, so timing of complications and antibiotic therapy for BSI could not be evaluated. In addition, many possible risk factors for sepsis, such as catheterization, time to the onset of feeding, and total parenteral nutrition had not been captured and were therefore not available for analysis.

## Conclusions

The present study confirms that there is an alarming increase in MDRE with the emergence of CRE. This confirms the urgent need to intensify antimicrobial stewardship efforts and address infection control and prevention in neonatal units in LMICs. In particular, the overuse of broad-spectrum antibiotics should be discouraged. In addition, other treatment and prevention modalities, such as strategies targeting the neonatal gut microbiome, should be investigated.

## Data Availability

Data will be made available upon reasonable request to the corresponding author.

## Abbreviations

BSI: Blood stream infection
CRE: Carbapenem resistant Enterobacteriaceae
EOS: Early onset sepsis
ESBL: Extended βlactamase
LMIC: low and middle income country
LOS: Late onset sepsis
MDRE: Multi drug resistant Enterobacteriaceae
MDRO: Multidrug resistant organisms
MIC: Minimum inhibitory concentration
NDM 1: New Delhi metallo β lactamase
NEC: Necrotizing enterocolitis
REDCAP: Research Electronic Data Capture
